# A history of gastrectomy is a risk factor for choledocholithiasis in patients undergoing cholecystectomy: A single center retrospective study

**DOI:** 10.1002/ags3.70008

**Published:** 2025-05-07

**Authors:** Yuki Matsui, Daisuke Hashimoto, So Yamaki, Kazuki Matsumura, Hidetaka Miyazaki, Yuji Ikeda, Denys Tsybulskyi, Thanh Sang Nguyen, Sohei Satoi

**Affiliations:** ^1^ Department of Pancreatobiliary Surgery Kansai Medical University Hirakata Japan; ^2^ Division of Surgical Oncology University of Colorado Anschutz Medical Campus Aurora Colorado USA

**Keywords:** choledocholithiasis, cholelithiasis, gastrectomy, prophylactic cholecystectomy

## Abstract

**Aim:**

The incidence of cholelithiasis after gastrectomy is higher than that in the general population; however, the incidence and risk factors for choledocholithiasis have not been well reported. We aimed to assess the association between a history of gastrectomy and choledocholithiasis.

**Methods:**

A total of 3025 patients who underwent cholecystectomy with or without choledocholithotomy between January 2006 and December 2020 at Kansai Medical University, Japan were included in this study. Patients were divided into a gastrectomy group with a history of gastrectomy (173 patients, 5.7%) and a control group having no history of gastrectomy (2852 patients, 94.3%).

**Results:**

The incidence of choledocholithiasis was 61.8% and 22.2% in the gastrectomy and control groups, respectively, with a significantly higher incidence in the gastrectomy group (*p* < 0.0001). Multivariate analysis showed that age, sex, history of gastrectomy, and previous surgery except gastrectomy were risk factors for the development of choledocholithiasis, with a history of gastrectomy being the strongest risk factor (Odds Ratio 3.78, 95% Confidence Interval 2.71–5.27). The incidence values of choledocholithiasis in the Billroth I, Billroth II, and Roux‐en‐Y methods were 44.7%, 70.6%, and 69.7%, respectively, and were significantly lower in the Billroth I group than in the Roux‐en‐Y group (*p* = 0.009). The median time from gastrectomy to development of choledocholithiasis was 5.5 years for Roux‐en‐Y, which was significantly faster than 20 years for Billroth I and 35 years for Billroth II.

**Conclusion:**

Gastrectomy is a known risk factor for choledocholithiasis. Concomitant cholecystectomy during gastrectomy may be indicated in older men.

## Graphical abstract

This retrospective study suggests that gastrectomy is a risk factor for choledocholithiasis. Especially in terms of the differences in reconstruction methods, the median time from gastrectomy to development of choledocholithiasis was 5.5 years for Roux‐en‐Y, which was significantly faster than 20 years for Billroth I and 35 years for Billroth II.

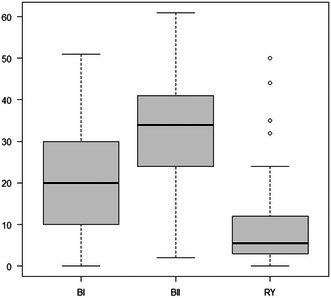

## 1 INTRODUCTION

The prevalence of cholelithiasis in Japan is estimated to be approximately 5%–10%^1^, ^2^ and approximately 5% of these patients progress to cholecystitis.^3^, ^4^, ^5^ Among them, those with a history of gastrectomy exhibit a higher incidence of cholelithiasis (ranging from 6.5% to 25%) compared with the general population.^3^, ^4^, ^6^, ^7^ It has also been reported^8^ that 8%–20% of patients with cholelithiasis suffer from choledocholithiasis, and in general, patients with cholelithiasis are at risk of choledocholithiasis. However, studies on the incidence and risk factors for choledocholithiasis after gastrectomy are limited. The Japanese guidelines for cholelithiasis suggest that cholecystectomy for asymptomatic cholecystolithiasis is of little significance. However, asymptomatic choledocholithiasis carries the risk of developing cholangitis or other problems, suggesting that stone removal should be performed.^1^, ^9^ It has been reported that as many as 11.6%^10^ of all cases of acute cholangitis can be categorized as grade III (severe) of the Tokyo Guidelines 2018 (TG18)^11^ criteria for severity, and cholangitis is a very serious condition. The mortality rate of acute cholangitis is approximately 10%, compared with a mortality rate of ~1% for acute cholecystitis, indicating that it is a dangerous condition.^11^ Therefore, it is important to investigate the incidence and risk factors for choledocholithiasis after gastrectomy.

The aim of this study was to determine whether a history of gastrectomy is a risk factor in the development of cholangitis in patients who underwent cholecystectomy.

## 2 METHODS

### 2.1 Patients and study design

**FIGURE 1:**
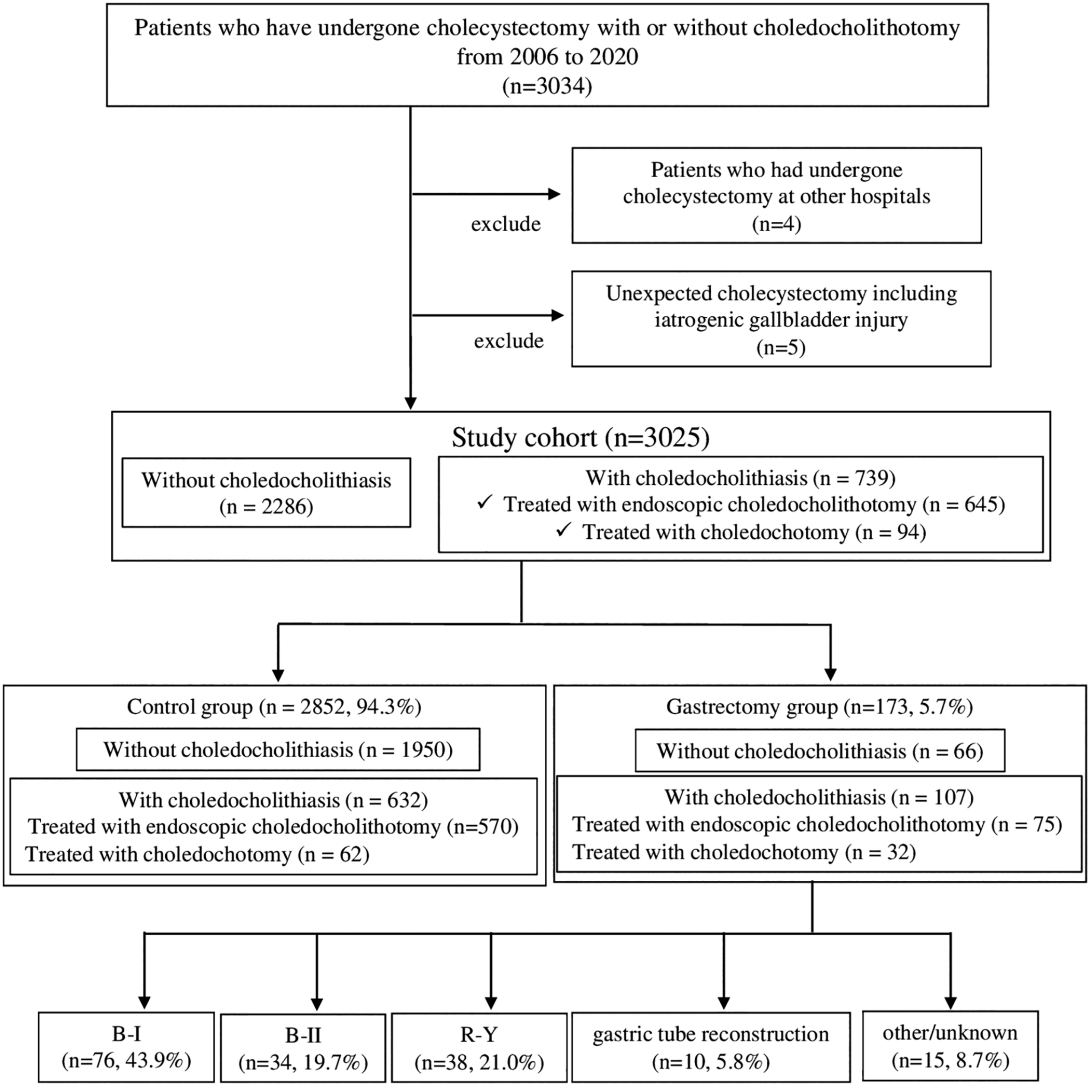
Study algorithm. Control group, cohort without a history of gastrectomy; Gastrectomy group, cohort with a history of gastrectomy; B‐I, Billroth I; B‐II, Billroth II; R‐Y, Roux‐en Y.

This single‐center retrospective study included 3025 patients who underwent cholecystectomy with or without choledocholithotomy at Kansai Medical University Hospital, Japan, between January 2006 and December 2020 (Figure 1). Patients who had undergone cholecystectomy at other hospitals and patients who underwent unexpected cholecystectomy, such as cholecystectomy for intraoperative iatrogenic gallbladder injury, were excluded. We divided our patient cohort into the gastrectomy group with a history of gastrectomy (*n* = 173, 5.7%) and the control group without a history of gastrectomy (*n* = 2852, 94.3%). The incidence of choledocholithiasis within these two groups was compared, and risk factors were scrutinized. The gastrectomy group was further subdivided according to the method of reconstruction, and differences in time to development of choledocholithiasis as well as incidence with respect to method of reconstruction were investigated. A patient with a stone in the common bile duct was diagnosed with choledocholithiasis based on magnetic resonance cholangiopancreatography (MRCP). There were 739 cases of patients diagnosed with choledocholithiasis. Of these, 645 (87.3%) underwent cholecystectomy after endoscopic choledocholithotomy and 94 (12.7%) with choledochotomy. At our hospital, cholecystectomy was either performed in cases diagnosed with acute cholecystitis in accordance with the TG18^11^ or in cases of symptomatic cholelithiasis.

### 2.2 Statistical analysis

The difference in the incidence of choledocholithiasis between the gastrectomy and control groups was evaluated by the chi‐square test. Univariate and multivariate analyses employing the Cox proportional hazards model were used to assess the risk factors for choledocholithiasis. Among the clinical variables included in the univariate analysis, those with a two‐sided *p* value of less than 0.1 were chosen for multivariate analysis with stepwise selection, where a two‐sided *p* value of less than 0.05 was considered to indicate a statistically significant difference. The cutoff value for age was calculated from the Receiver Operating Characteristic (ROC) curve using Youden's index. All statistical analyses were performed using the EZR software version 1.61.^12^

## 3 RESULTS

### 3.1 Patient characteristics

The clinical characteristics of the study cohort (*n* = 3025) are summarized in Table 1. A significantly higher proportion of men and the elderly were found in the gastrectomy group, relative to the control group (*p* < 0.0001). Laparoscopic cholecystectomy was performed in 63% of patients in the gastrectomy group, and this was significantly lower than 92.1% of such patients in the control group (*p* < 0.0001). Elective cholecystectomy accounted for 74% of patients in the gastrectomy group, and this was significantly lower than 85.5% of such patients in the control group (*p* < 0.0001). The incidence of choledocholithiasis was 24.4% in the total cohort, 61.8% in the gastrectomy group, and 22.2% in the control group, reflecting a very high incidence in the gastrectomy group (*p* < 0.0001). Most of the patients with choledocholithiasis were successfully treated with endoscopic choledocholithotomy. Choledochotomy was performed in 29.9% of the gastrectomy group. The median value for operation time was 70 min in the total cohort, 122 min in the gastrectomy group, and 68 min in the control group. The median value for postoperative hospital stay was 3 days in the total cohort, 5 days in the gastrectomy group, and 3 days in the control group. All the above data were significantly different in comparisons between any two groups (*p* < 0.0001).

**TABLE 1:** Clinical characteristics of patients.

	Total (*n* = 3025)	Gastrectomy group (*n* = 173)	Control group (*n* = 2852)	*p* Value
Age	65 (4–95)	74 (48–88)	64 (4–95)	<0.0001
Sex				
Male (%)	1469 (48.5)	142 (82.1)	1327 (48.1)	<0.0001
Female (%)	1556 (51.5)	31 (17.9)	1525 (51.9)	
Previous surgery except gastrectomy (%)	263 (8.7)	20 (11.6)	243 (8.5)	0.1681
Laparoscopic cholecystectomy (%)	2735 (90.4)	109 (63)	2626 (92.1)	<0.0001
Open surgery (%)	290 (9.6)	64 (47)	226 (7.9)	
Elective cholecystectomy (%)	2566 (84.8)	128 (74.0)	2438 (85.5)	<0.0001
Emergent cholecystectomy (%)	459 (15.2)	45 (26.0)	414 (14.5)	
Choledocholithiasis				
Presence/absence (%)	739 (24.4)/2282	107 (61.8)/66	632 (22.2)/2220	<0.0001
Treatment for choledocholithiasis				
Endoscopic choledocholithotomy (%)	645 (87.3)	75 (70.1)	570 (90.2)	<0.0001
Choledochotomy (%)	94 (12.7)	32 (29.9)	62 (9.8)	
Operation time for cholecystectomy, min	70 (19–515)	122 (46–311)	68 (19–515)	<0.0001
Postoperative hospital stay, days	3 (1–230)	5 (3–69)	3 (1–230)	<0.0001

*Note*: Age, operation time and postoperative hospital stay are each expressed as a median (range).

Abbreviation: ERCP, endoscopic retrograde cholangiopancreatography.

### 3.2 Gastrectomy group diseases and reconstruction methods

Details of the gastrectomy group are shown in Table 2. The most common primary diseases of gastrectomy were gastric cancer in 64.7% of patients, followed by ulcer, esophageal cancer, neuroendocrine tumor, and gastrointestinal stromal tumor. Roux‐en Y (R‐Y) reconstruction was the most common method after gastrectomy in 43.9% of patients, followed by Billroth‐I (B‐I), Billroth‐II (B‐II), and gastric tube reconstruction after esophagectomy.

**TABLE 2:** Details of gastrectomy and reconstruction methods.

	*N* = 173
Primary disease (%)	
Gastric cancer	112 (64.7)
Ulcer	46 (26.6)
Esophageal cancer	10 (5.78)
NET	1 (0.58)
GIST	1 (0.58)
Other/unknown	3 (1.73)
Reconstruction method (%)	
Roux‐en Y	76 (43.9)
Billroth I	38 (21.0)
Billroth II	34 (19.7)
Gastric tube reconstruction	10 (5.78)
Other/unknown	15 (8.67)
Operative method of gastrectomy	
Distal gastrectomy	119 (68.8)
Total gastrectomy	33 (19.1)
Proximal gastrectomy	4 (2.31)
Pylorus preserving gastrectomy	1 (0.58)
Local resection	3 (1.73)
Other/unknown	13 (7.51)

Abbreviations: GIST, gastrointestinal stromal tumor; NET, neuroendocrine tumor.

### 3.3 Univariate and multivariate analyses regarding risk factors for choledocholithiasis

As shown in Table 3, there were significant differences in age, being male, history of gastrectomy, and previous surgery except gastrectomy, when we compared patients with and without choledocholithiasis. In addition, Table 4 shows that being 65 years of age or older, being male, having a history of gastrectomy and previous surgery except gastrectomy were all risk factors according to the results of our multivariate analysis. Of these four categories, having a history of gastrectomy was found to be the strongest risk factor for choledocholithiasis, with an Odds Ratio of 3.780 (95% Confidence Interval 2.710–5.270).

**TABLE 3:** Characteristics of patients by presence or absence of choledocholithiasis.

	Choledocholithiasis		*p* Value
	Presence (*n* = 739)	Absence (*n* = 2286)	
Age (range)	71 (11–91)	63 (4–95)	<0.0001
Sex male/female (%)	426 (57.6)/313 (42.4)	1043 (45.6)/1022 (54.4)	<0.0001
Laparoscopic/Open (%)	126 (17.1)/613 (82.9)	2122 (93.0)/164 (7.1)	<0.0001
Elective/Emergent (%)	646 (87.4)/93 (12.6)	1920 (84.0)/366 (16.0)	0.024
Operation time, min (range)	80 (19–396)	67 (21–515)	<0.0001
Postoperative hospital stay, days (range)	4 (1–58)	3 (1–230)	<0.0001
History of gastrectomy	69 (11.9%)	35 (1.95%)	<0.0001
Previous surgery except gastrectomy	81 (11.0%)	159 (6.95%)	<0.0005

*Note*: Previous surgery except gastrectomy: Includes all surgeries other than gastrectomy, from surgery for cancer such as colorectal cancer to surgery for benign conditions such as hernia.

**TABLE 4:** Univariate and multivariate analysis for risk factors of choledocholithiasis.

	Univariate analysis		Multivariate analysis	
	*p* Value	OR (95% CI)	*p* Value	OR (95% CI)
Age ≥65	<0.0001	2.820 (2.37–3.36)	<0.0001	2.390 (2.000–2.860)
Sex; male	<0.0001	1.620 (1.370–1.920)	<0.0001	1.390 (1.170–1.660)
History of gastrectomy	<0.0001	5.690 (4.140–7.840)	<0.0001	3.780 (2.710–5.270)
Previous surgery except gastrectomy	<0.0001	1.890 (1.450–2.470)	0.0009	1.600 (1.210–2.120)

Abbreviations: 95% CI, 95% confidence interval; OR, odds ratio.

### 3.4 Development of choledocholithiasis and time interval between gastrectomy and development of choledocholithiasis according to procedure of reconstruction

**FIGURE 2:**
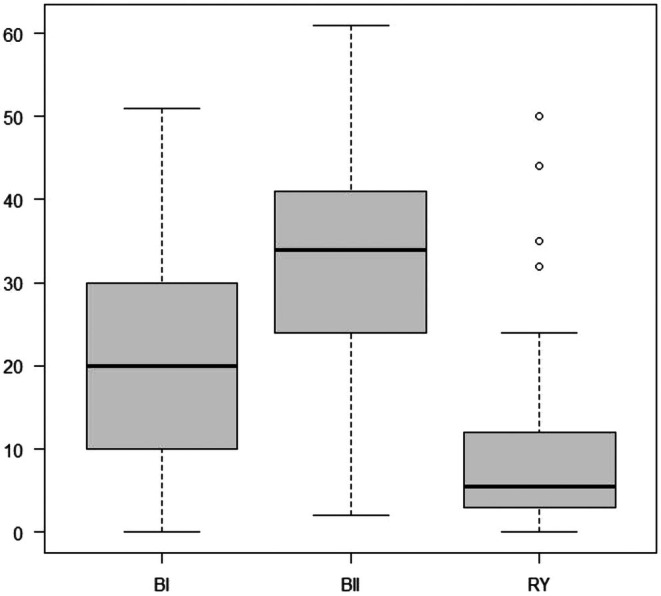
Duration from gastrectomy to development of choledocholithiasis. Kruskal–Wallis test: *p* value < 0.0001. B‐I: Billroth I method with a choledocholithiasis development duration median value of 20 years, range 0–51 years. B‐II: Billroth II method with a choledocholithiasis development duration median value of 34 years, range 2–61 years. R‐Y: Roux‐en Y method with a choledocholithiasis development duration median value of 5.5 years, range 0–50 years. Steel‐Dwass test: B‐I versus B‐II, *p* = 0.002. B‐I versus R‐Y, *p* < 0.0001. B‐II versus R‐Y, *p* < 0.0001.

The incidence of choledocholithiasis in the B‐I, B‐II, and R‐Y methods were 44.7%, 70.6%, and 69.7%, respectively. After applying the Bonferroni correction, this value became significantly lower for patients who underwent B‐I compared to those who were treated via the R‐Y technique (*p* = 0.009) (Table 5). There was no significant difference in choledocholithiasis incidence levels between patients treated with B‐I versus B‐II, or between those who underwent B‐II versus R‐Y surgeries. Figure 2 shows the duration from gastrectomy to choledocholithiasis development categorized by reconstruction method, with median values of 20, 34, and 5.5 years for B‐I, B‐II, and R‐Y methods, respectively. The duration till choledocholithiasis development was significantly shorter in patients treated via the R‐Y method compared to those treated by the other two techniques.

**TABLE 5:** Incidence of choledocholithiasis compared by procedure of reconstruction.

	Billroth I (*n* = 38)	Billroth II (*n* = 34)	Roux‐en Y (*n* = 76)	*p* Value
Choledocholithiasis (%)				
Presence	17 (44.7)	24 (70.6)	53 (69.7)	
Absence	21 (55.3)	10 (29.4)	23 (30.3)	
Billroth I vs. Billroth II				0.032
Billroth I vs. Roux‐en Y				0.009
Billroth II vs. Roux‐en Y				0.931

*Note*: Bonferroni's correction sets the significance level at 0.0167 or less.

## 4 DISCUSSION

It has been recently estimated that approximately 5%–10% of the general population in Japan has cholelithiasis^1^, ^2^ and that 60%–80% of patients with cholelithiasis have no symptoms.^13^ However, acute cholecystitis is the most frequent complication of cholelithiasis and requires attention. It has also been reported that 10%–20% of patients undergoing cholecystectomy for symptomatic cholecystitis had complicated choledocholithiasis.^14^ Evidence‐based Clinical Practice Guidelines for Cholelithiasis 2021 from the Japanese Society of Gastroenterology recommend follow‐up for asymptomatic cholelithiasis, while removal is recommended for asymptomatic choledocholithiasis.^1^ This is because 11.6% of cases of acute cholangitis account for grade III (severe) of the TG18 severity scale and the mortality rate of 10% is high compared to 1% for acute cholecystitis.^11^ Choledocholithiasis, even if asymptomatic, is at risk of becoming severe and fatal due to complications of acute cholangitis or acute pancreatitis.^15^ This implies that it is critical to investigate the incidence and risk factors for choledocholithiasis in patients who underwent cholecystectomy after gastrectomy. The incidence of cholelithiasis in patients with a history of gastrectomy has been noted to be higher than in the general population, ranging from 6.5% to 25%.^3^, ^4^, ^6^, ^7^ Based on these reports, an increased incidence of choledocholithiasis in patients who underwent cholecystectomy after gastrectomy was expected and has provided the rationale for the present study. Our present findings showed that a total of 61.8% of patients in the gastrectomy group had choledocholithiasis, which was significantly higher than 22.2% in the control group. The prevalence of choledocholithiasis in the control group of our patient cohort was 22.2%, which does not deviate significantly from the prevalence reported by Joyce et al.^14^

Various factors have been suggested as causes of the increase in cholelithiasis incidence among patients who underwent cholecystectomy after gastrectomy, and impaired gallbladder motility due to vagotomy is a commonly known factor. Especially in cases of total gastrectomy, both vagus nerves in the anterior and posterior hepatic plexus are damaged, leading to a high incidence of cholelithiasis.^4^ According to our current data, as shown in Table 2, distal gastrectomy was the most common type of gastrectomy (119 cases) in our patient cohort, followed by total gastrectomy (33 cases). The incidence of choledocholithiasis after distal gastrectomy was 62.2%, and that after total gastrectomy was 69.7%. Although these two results are not significantly different, the incidence of choledocholithiasis tended to be higher after total gastrectomy in our data, confirming the results reported by Kobayashi et al.^4^ Gastrectomy and gastrointestinal reconstruction increase the incidence of cholelithiasis by causing biliary stasis through the sphincter of Oddi, reflux infection,^16^ and changes in cholecystokinin secretion.^17^ The impact of the method of reconstruction on the incidence of cholelithiasis in patients who underwent cholecystectomy after gastrectomy is still debated. The R‐Y method has been reported to significantly increase cholelithiasis incidence after distal gastrectomy compared with the B‐I technique.^4^, ^18^, ^19^, ^20^ Our present study on choledocholithiasis has also shown the same results as reported by earlier studies on cholelithiasis, with R‐Y and B‐II methods significantly increasing the incidence of choledocholithiasis compared with the B‐I technique. Moreover, R‐Y was associated with a markedly earlier onset of choledocholithiasis compared with other reconstruction methods. Compared with B‐I, in the R‐Y technique, food does not pass through the duodenum, and the passage route is non‐physiological. For this reason, in patients undergoing R‐Y surgery, the secretion of cholecystokinin is insufficient, and it is thought that cholelithiasis is likely to occur due to the accumulation of bile. In addition, it has been suggested that R‐Y surgery causes an increase in duodenal intraluminal pressure compared with the B‐I technique, and that retrograde infection of the biliary tract is likely to occur.^21^ In addition, R‐Y method was associated with earlier onset of choledocholithiasis than B‐II method in this study, although this difference was not statistically significant. In B‐II method, a side‐to‐side jejunostomy (so‐called Braun anastomosis) is generally added. We hypothesize that duodenal lumen pressure may play a role in the development of choledocholithiasis after gastrectomy, and the duodenal intraluminal pressure tends to be decreased due to the Braun anastomosis in B‐II method, but not in R‐ Y. However, there were no reports comparing R‐Y and B‐II methods directly. Based on the above reports and the results of our present study, we reason that R‐Y surgery may be associated with a higher incidence and earlier onset of choledocholithiasis than B‐I. In summary, post‐gastrectomy patients who underwent R‐Y surgery should be more vigilant against choledocholithiasis.

Since acute cholecystitis, choledocholithiasis, and pancreatitis can sometimes lead to severe conditions in patients, there are scattered references recommending the use of prophylactic cholecystectomy during gastric cancer surgery for asymptomatic cholelithiasis.^19^, ^21^ On the other hand, the CHOLEGAS trial, a multicenter randomized controlled trial conducted in Italy, concluded that prophylactic cholecystectomy in patients without cholelithiasis at the time of gastric cancer surgery did not significantly improve postoperative outcomes.^22^ Further, a large cohort analysis of cholelithiasis in 17 325 patients undergoing gastric cancer surgery in Taiwan found that only 560 (3.2%) post‐gastrectomy patients required subsequent cholecystectomy. The authors of that study concluded that prophylactic cholecystectomy for patients without cholelithiasis should be based on patient characteristics and surgeon judgment.^20^ As a result of the above, cholecystectomy is not routinely performed at the time of gastrectomy in mainstream clinical practice in Japan. However, it is suggested that simultaneous cholecystectomy may reduce the incidence of choledocholithiasis in patients where at least asymptomatic cholelithiasis or biliary sludge is noted at the time of gastrectomy. This option is critical when considering disease severity and the high mortality rate of acute cholangitis. In this study, 61.8% of patients who underwent cholecystectomy and had a history of gastrectomy developed choledocholithiasis. When contemplating cholecystectomy for patients with asymptomatic cholelithiasis or biliary sludge at the time of gastrectomy, the surgeon should consider the risk of postoperative cholecystitis as well as the incidence of choledocholithiasis. Especially, the older male (65 years or older) patient should undergo cholecystectomy because a previous report^23^ has shown a higher incidence of choledocholithiasis and an elevated mortality rate in patients who were 75 years of age or older.

This study has several limitations. It is a single‐center, retrospective study, and the patient cohort is composed of patients who underwent cholecystectomy with or without choledocholithotomy. However, the prevalence of choledocholithiasis in the control group of our cohort was 22.2%, which does not deviate significantly from the 10% to 20% reported by Joyce et al.^14^

In conclusion, our findings suggest that gastrectomy is a risk factor for choledocholithiasis. Compared to the B‐I method, the R‐Y technique was associated with a significantly earlier onset and significantly increased incidence of choledocholithiasis. We recommend that the incidence of cholecystitis as well as choledocholithiasis should be considered when deciding on cholecystectomy at the time of gastrectomy.

## AUTHOR CONTRIBUTIONS

**Yuki Matsui:** Conceptualization; data curation; formal analysis; investigation; methodology; project administration; resources; software; validation; visualization; writing – original draft. **Daisuke Hashimoto:** Conceptualization; data curation; formal analysis; investigation; methodology; project administration; resources; software; supervision; validation; writing – review and editing. **So Yamaki:** Data curation; formal analysis; investigation; methodology. **Kazuki Matsumura:** Formal analysis; investigation; methodology; software. **Hidetaka Miyazaki:** Formal analysis; investigation; methodology. **Yuji Ikeda:** Formal analysis; investigation; methodology. **Denys Tsybulskyi:** Formal analysis; investigation. **Thanh Sang Nguyen:** Investigation; methodology. **Sohei Satoi:** Conceptualization; data curation; formal analysis; investigation; methodology; project administration; resources; software; supervision; validation; visualization; writing – review and editing.

## FUNDING INFORMATION

The authors did not receive support from any organization for the submitted work.

## CONFLICT OF INTEREST STATEMENT

S.S. received research funding from Nihon Servier, Amino‐Up Co., and Boston Scientific.

## ETHICS STATEMENT

Approval of the research protocol by an Institutional Reviewer Board: This study was reviewed and approved (Approval Number: 2020131) by the Institutional Review Board of Kansai Medical University, Japan, and complied with the STROBE guidelines.^24^ All the procedures in this study were performed in accordance with the guidelines of the Declaration of Helsinki.

Informed Consent: N/A.

Registry and the Registration No. of the study/trial: N/A.

Animal Studies: N/A.
